# Comparison of the PEEK cage and an autologous cage made from the lumbar spinous process and laminae in posterior lumbar interbody fusion

**DOI:** 10.1186/s12891-016-1237-y

**Published:** 2016-08-30

**Authors:** Bin Lin, Hui Yu, Zhida Chen, Zhuanzhi Huang, Wenbin Zhang

**Affiliations:** Department of Orthopaedics, the 175th Hospital of PLA, Southeast Hospital of Xiamen University, 269 Zhanghua Road, Zhangzhou, Fujian 363000 People’s Republic of China

**Keywords:** Posterior lumbar interbody fusion (PLIF), PEEK cage, Spinous process, Lumbar degenerative disease

## Abstract

**Background:**

A prospective cohort study was performed to evaluate the clinical and radiological outcomes following posterior lumbar interbody fusion (PLIF) in patients treated with a PEEK cage compared to those treated with an autologous cage using the lumbar spinous process and laminae (ACSP).

**Methods:**

Sixty-nine consecutive patients with lumbar degenerative disc disease were randomly assigned to either a PEEK cage (group A, n = 34) or an ACSP (group B, n = 35). Monosegmental PLIF was performed in all patients. Mean lumbar lordosis, mean disc height, visual analog scale (VAS) scores, functional outcomes, fusion rates and complication rates were recorded and compared. The patients were followed postoperatively for a minimum of 2 years.

**Results:**

Successful radiographic fusion was documented in all patients. No flexion–extension hypermobility or pedicle screw loosening or breakage occurred during the follow-up period. No significant difference existed between the 2 groups when comparing the mean lumbar lordosis, mean disc height, visual analog scale (VAS) scores, functional outcomes, fusion rates or complication rates. Overall satisfactory results were achieved in both groups.

**Conclusions:**

The results suggest that the ACSP appears to be equally as safe and effective as the PEEK cage.

**Trial registration:**

ISRCTN25558534. Retrospectively registered 16/02/2016.

## Background

Lumbar degenerative disc disease is a common disease in adults [1], often causing low back pain and leg pain that frequently requires surgery [2]. It is hypothesized that lumbar degenerative disc disease begins with dehydration of the intervertebral disc accompanied by decreased tensile strength of the annulus fibrosus [3]. This is followed by a corresponding loss of disc height that can lead to posterior facet joint subluxation and to an abnormal pattern of motion that causes segmental instability [3]. For several decades, posterior lumbar interbody fusion (PLIF) has been considered standard of care for lumbar degenerative disc disease requiring surgery [4].

The advantages of PLIF include restoration of disc height, disc stabilization, nerve root decompression and reinforcement of the weight-bearing axis in the anterior segment of the spinal column [5, 6]. Traditionally, PLIF is performed using synthetic intervertebral cages with autogenous bone grafted from the corticocancellous iliac crest or from another bone graft material implanted in the intervertebral space [7]. The implants that are inserted into the intervertebral disc space come in various configurations [8, 9]. In recent years, cage implants designed with polyetheretherketone (PEEK) have been widely accepted with excellent clinical outcomes reported in the literature [10]. However, the nonresorbable property of the PEEK cage may potentially lead to risk of long-term complications and need for surgical reintervention for implant removal. Shortcomings of nonresorbable cage implants have been confirmed in several retrieval studies [11, 12]. In an effort to address the identified shortcomings of the traditional PEEK cage, we investigated an alternative cage made using an autologous graft from a lumbar spinous process and laminae by en bloc resection for use in PLIF. Previous reports have shown that high fusion rate was obtained in PLIF by using spinous processes and laminae at a single level [13]. Therefore, we hypothesize that the autologous cage from the lumbar spinous process and laminae (ACSP) will show similar clinical and radiological results to those obtained using a PEEK cage.

Currently, no studies exist that directly compare the performance of the PEEK cage and the ACSP. Currently, there is limited clinical experience with the application of the PEEK cage in lumbar spinal fusion. This study evaluates the clinical efficacy of PLIF with the PEEK cage and the ACSP in lumbar degenerative disc disease.

## Methods

### Patient population and randomization process

Sixty-nine consecutive patients (40 males, 29 females) who underwent posterior lumbar interbody fusion (PLIF) by a single surgeon (BL) and met inclusion criteria were prospectively enrolled between December 2008 and December 2010. Inclusion criteria included: age between 30 and 70 years; disc pathology requiring surgical intervention for decompression; one intended level of interbody fusion between L3 and S1; radiological evidence of instability, spondylolisthesis and the presence of degenerative stenosis, or symptomatic degenerative disc disease; and persistent or recurrent low back or leg pain lasting at least 6 months and resulting in a significant reduction of quality of life. Exclusion criteria included: need for two or more levels of fusion; active infection; metabolic disease; severe osteoporosis, symptomatic vascular disease; previous spinal surgery other than a lumbar discectomy in L3-L4, L4-L5, or L5-S1; any major psychological problem; the combination of degenerative scoliosis and degenerative or isthmic spondylolithesis; morbid obesity; and individuals who are smokers.

The average age of participants was 46.5 years (range, 30–60 years). Once a patient agreed to participate, informed consent was obtained. All patients were randomly assigned to receive either a PEEK cage (group A) or an ACSP (group B). Demographic and clinical data from each patient was recorded and are displayed in aggregate by study group in Table 1. No statistically significant difference existed between the groups with regards to age, sex, preoperative diagnosis, or number of fusion segments.

**Table 1:** Demographics of the subjects in the two groups

Demographics	Group A	Group B	*p*-value
No. of patients	35	34	NS
Age range, y	30–60	30–59	NS
No. of women/men	15/20	16/18	NS
BMI range, kg/m^2^	20–30	19–30	NS
Symptom duration range	9.5 months to 24 years	8.5 months to 24 years	NS
Operated level, No.			
L3-L4/L4-L5/L5-S1	7/19/9	7/17/10	NS

Abbreviation: *NS* not significant

*P* value was compared by chi-square test, except for age, which was compared by *t* test

Group A contained 20 males and 15 females with a mean age of 46.1 years (range 30–60 years). Preoperative clinical manifestations were intermittent claudication (n = 4), unilateral or bilateral lower limb pain (n = 27), and nerve dysfunction (n = 4). One-level fusion with a PEEK cage was performed in all patients in group A.

Group B contained 18 males and 16 females with a mean patient age of 46.8 years (range 41–59 years). Preoperative clinical manifestations were intermittent claudication (n = 6), unilateral or bilateral lower limb pain (n = 23), and nerve dysfunction (n = 5). One-level fusion with an ACSP was performed in all patients in group B.

### Surgical technique

**Fig. 1:**
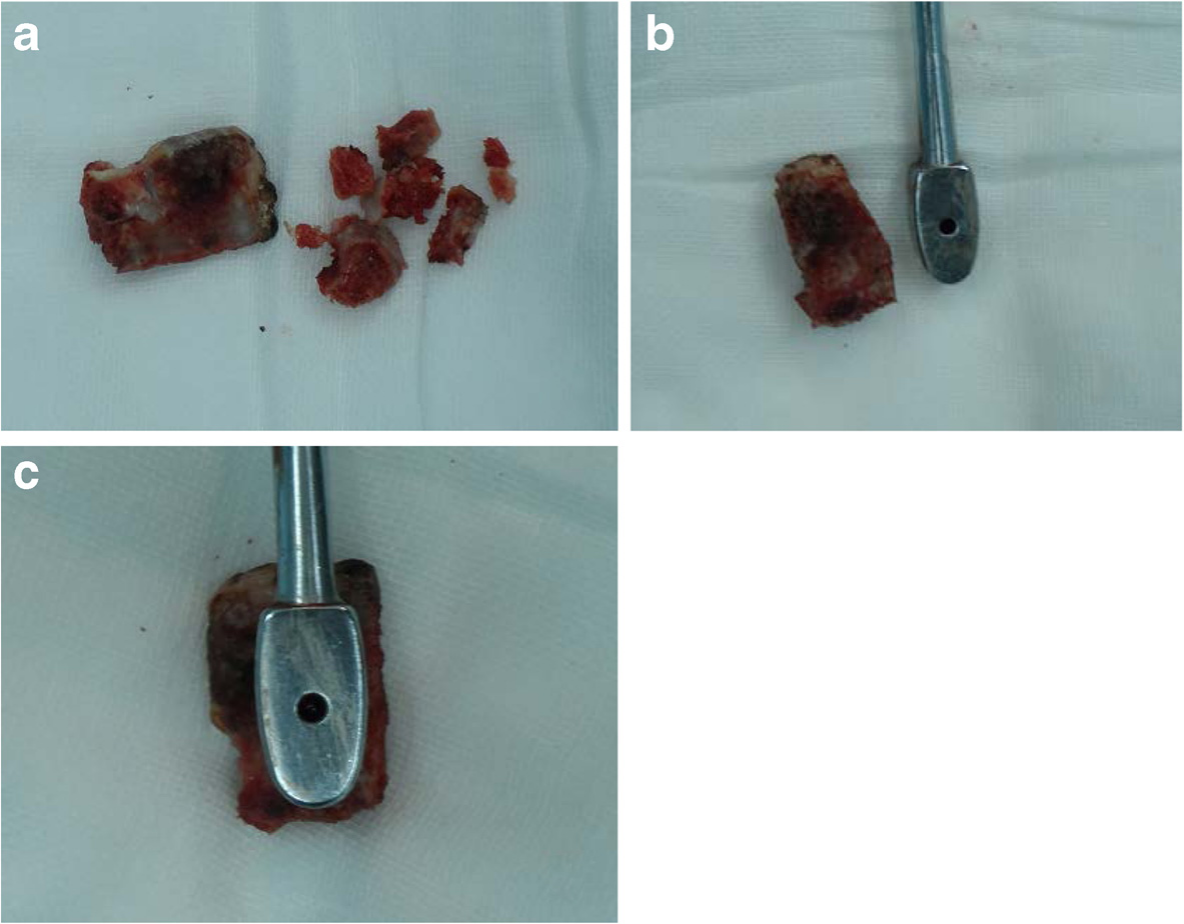
A series of images showing the preparation the natural cage: bone block harvested from the excised spinous process was trimmed into a natural cage and the blocks from the laminae were cut into small pieces (**a**). Images showing the comparison of the natural cage and a cage model (**b.c**)

All patients were given 2.0 g of cefazolin half an hour prior to surgery. Patients were placed in the prone position. General anesthesia was used. The bilateral paravertebral muscles were split and retracted laterally to the outer edge of the facet joint. After exposing the spinous process and both laminae, the surrounding soft tissues (e.g. ligamentum flavum and interspinous ligaments) were removed. En bloc resection of the spinous process and laminae was performed using an osteotome. Next, the entire nerve root and intervertebral space were exposed. The dura and nerve roots were protected with a nerve-root retractor. Adequate decompression was accomplished until subchondral bleeding bone was seen. After extensive discectomy, the endplate cartilage was removed, and measurements were taken using trial implants to find the appropriate size implant. In group A, a PEEK cage was inserted and impacted into the intervertebral space. In group B, a bone block was harvested from the excised spinous process and trimmed into an ACSP (Fig. 1), while the bone blocks from the laminae were cut into small pieces. The ACSP was inserted into the intervertebral space and the smaller laminar pieces were inserted and impacted surrounding the ACSP with a bonegrafting funnel. Finally, interpedicular screws were inserted with rods connected with/without cross-link devices.

### Postoperative management

All patients receive postoperative intravenous antibiotic for two days. Drainage was maintained for 48 h following surgery. In the week following the operation, patients were permitted to mobilize with metal lumbosacral support. However, substantial rotation and load bearing were forbidden.

### Clinical evaluation

All patients were scheduled for follow-up at 3, 6, 12 and 24 months postoperatively and annually thereafter. Visual Analogue Score (VAS) was obtained for low back pain both pre- and postoperatively at 2-year follow-up; functional outcome was assessed post-operatively using the Kirkaldy–Willis criteria [14] (Table 2).

**Table 2:** Kirkaldy-Willis Criteria: the modified criteria for functional outcome

	The modified Kirkaldy-Willis Criteria
Grade	Description
Excellent	The patient has returned to their normal work and other activities with little or no complaint.
Good	The patient has returned to their normal work but may have some restriction in other activities, and may on occasion after heavy work have recurrent back pain requiring a rest for a few days.
Fair	The patient has to reduce their working capacity, taking a lighter job or work part-time, and may occasionally have recurrence of pain requiring absence from work for one to two weeks, once or twice a year.
Poor	The patient does not return to work.

**Fig. 2:**
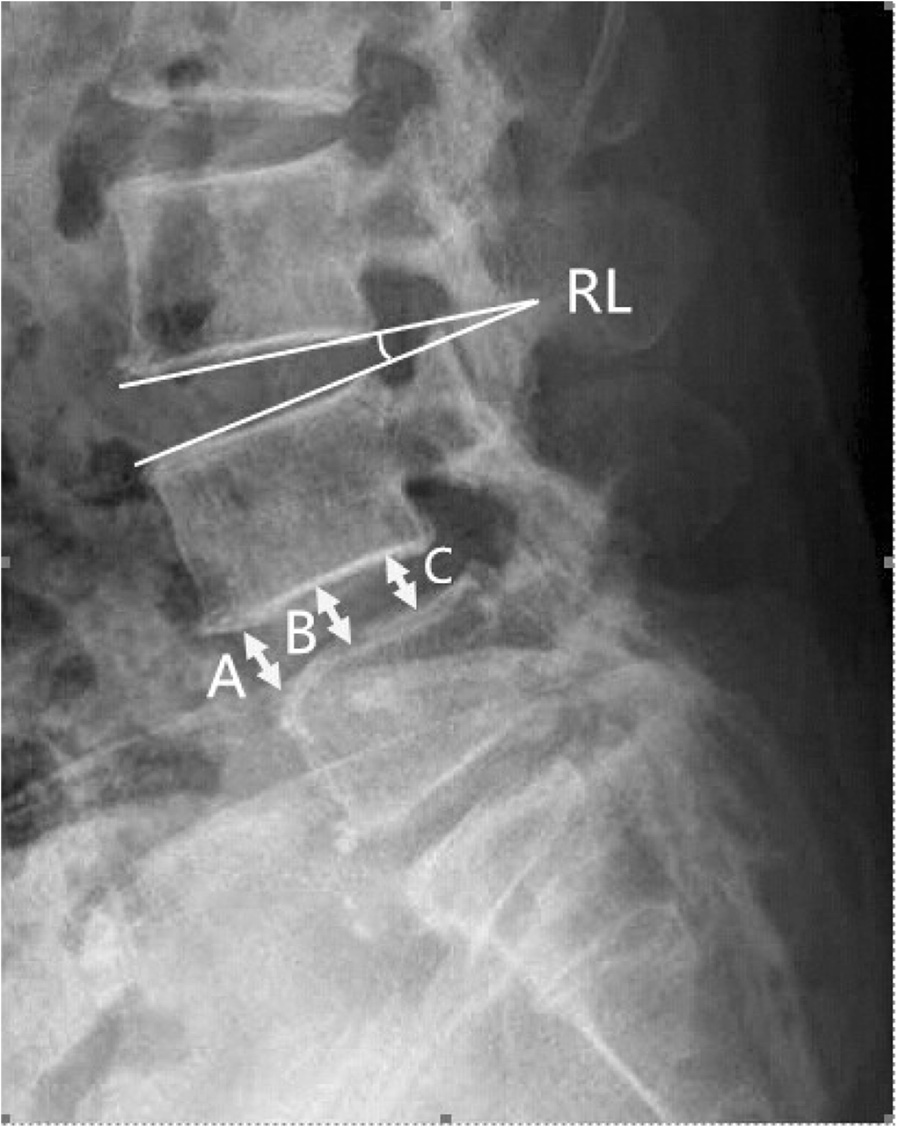
Radiographic measurements of the lumbar disc height: A anterior disc height, B middle disc height, C posterior disc height. Disc height = (A + B + C)/3 (mm). Measurement of regional lordosis (RL): the angle between the upper and lower edges of the intervertebral disc

Radiological assessment was recorded at each follow-up visit. Fusion status was evaluated by anteroposterior and lateral flexion and extension radiographs. Levels were regarded as solidly fused if radiographic evidence existed of bone bridging the disk space without lucency and the motion between the fused segments was less than 4° on flexion and extension views. More than 4° of motion or the presence of translation was considered a failure of fusion [15, 16]. Thin-section, high-resolution helical computed tomography was used annually to more accurately evaluate the bony trabeculae in the disk space. All images were independently reviewed by two experienced radiologists who were blinded to the clinical outcome and had not taken part in any other stage of the study. The disc height (DH) was calculated as the mean of the anterior, middle and posterior disc heights, and the sagittal diameter of the lower vertebral body from the anterior to posterior margin was measured at the midvertebral level (Fig. 2). The angle between the upper and lower edges of the intervertebral disc was defined as regional lordosis (RL) (Fig. 2). The data were analyzed independently by two clinicians.

### Statistical analysis

Statistical analysis was performed with SPSS 13.0 (SPSS, Inc., Chicago, IL, USA) for Windows. Population data were compared by chi-square test with the exception of age, which was compared by *t*-test. The data regarding RL and DH before surgery and at the time of final follow-up were analyzed by Student’s *t*-test. Preoperative and 2-year postoperative VAS scores were compared by *t* test. A *p*-value of less than 0.05 was considered to be significant.

## Results

All patients successfully underwent posterior lumbar interbody fusion (PLIF) with PEEK cages or ACSPs. All 69 patients had relief of pain at 2-year follow-up. The mean VAS scores decreased preoperatively to postoperatively in both groups; this difference was significant (*P* < 0.05). However, no significant differences in preoperative or postoperative VAS scores existed between the two groups (Table 3).

**Table 3:** The VAS Scores in the two groups

	Preoperative	Postoperative
Group A	7.23 ± 0.88	1.86 ± 0.63
Group B	7.54 ± 1.24	2.05 ± 0.61
*p*-value	NS	NS

Abbreviation: *NS* not significant

Data presented as mean ± SD. *P*-value was compared by Mann–Whitney *U*-test. Significant differences existed between pre- and postoperative VAS scores in both groups (*P* < 0.05); no significant difference existed between the 2 groups (*p* > 0.05)

Functional outcome was evaluated at a mean follow-up of 18.8 months (range, 12–24 months). There were no poor outcomes in either group. For group A, outcomes were excellent in 18 patients (51.4 %), good in 12 patients (34.3 %) and fair in 5 patients (14.3 %). For group B, outcomes were excellent in 19 patients (55.9 %), good in 11 patients (32.3 %) and fair in 4 patients (11.8 %). No significant differences were observed between the groups (Table 4).

**Table 4:** Functional outcome of the two groups

	Excellent	Good	Fair	Poor
Group A	18 (51.4 %)	12 (34.3 %)	5 (14.3 %)	0
Group B	19 (55.9 %)	11 (32.3 %)	4 (11.8 %)	0
*p*-value	NS	NS	NS	NS

Abbreviation: *NS* not significant

Data were compared by chi-square test. No significant difference existed between the two groups (*p* > 0.05)

Between 8 and 12 weeks postoperatively, fusion had occurred in 32 patients (94.1 %) in group A and in 34 patients (97.1 %) in group B. All remaining patients achieved successful fusion by 24 months, and there was no significant difference in fusion rates between the two groups. The mean lumbar lordosis (Table 5) and intervertebral disc height (DH) (Table 6) were restored and preserved. In group A, four patients (11.4 %) had a dural tear while three patients (8.8 %) had this complication in group B. All dural tears were repaired at the time of surgery using a fat pad. One patient in group A (2.9 %) had a superficial wound infection, which responded to a short course of oral antibiotics. There were no deep infections in either group.

**Table 5:** Regional Lordosis (°) of the two groups

	Group A		Group B		*p*-value
	Pre-OP	Post-OP	Pre-OP	Post-OP	
L3/4	10.86 ± 3.63	18.86 ± 2.91	10.29 ± 3.30	20.00 ± 2.65	NS
L4/5	10.88 ± 3.60	19.53 ± 2.84	11.33 ± 3.09	20.50 ± 3.88	NS
L5/S1	11.67 ± 3.87	23.67 ± 2.50	12.10 ± 3.48	22.00 ± 4.77	NS

Abbreviation: *NS* not significant

Data presented as mean ± SD. *P*-value was compared by Student’s *t*-test. Significant differences existed between pre- and postoperative regional lordosis in both groups (*P* < 0.05); no significant difference existed between the two groups (*p* > 0.05)

**Table 6:** Disc height (mm) of the two groups

	Preoperative	Postoperative
Group A	21.91 ± 3.75	45.43 ± 3.51
Group B	21.50 ± 4.77	43.29 ± 3.62
*p*-value	NS	NS

Abbreviation: *NS* not significant

Data presented as mean ± SD. *P* value was compared by Student’s *t*-test. Significant differences existed between pre- and postoperative disc height in both groups (*P* < 0.05); no significant difference existed between the two groups (*p* > 0.05)

**Fig. 3:**
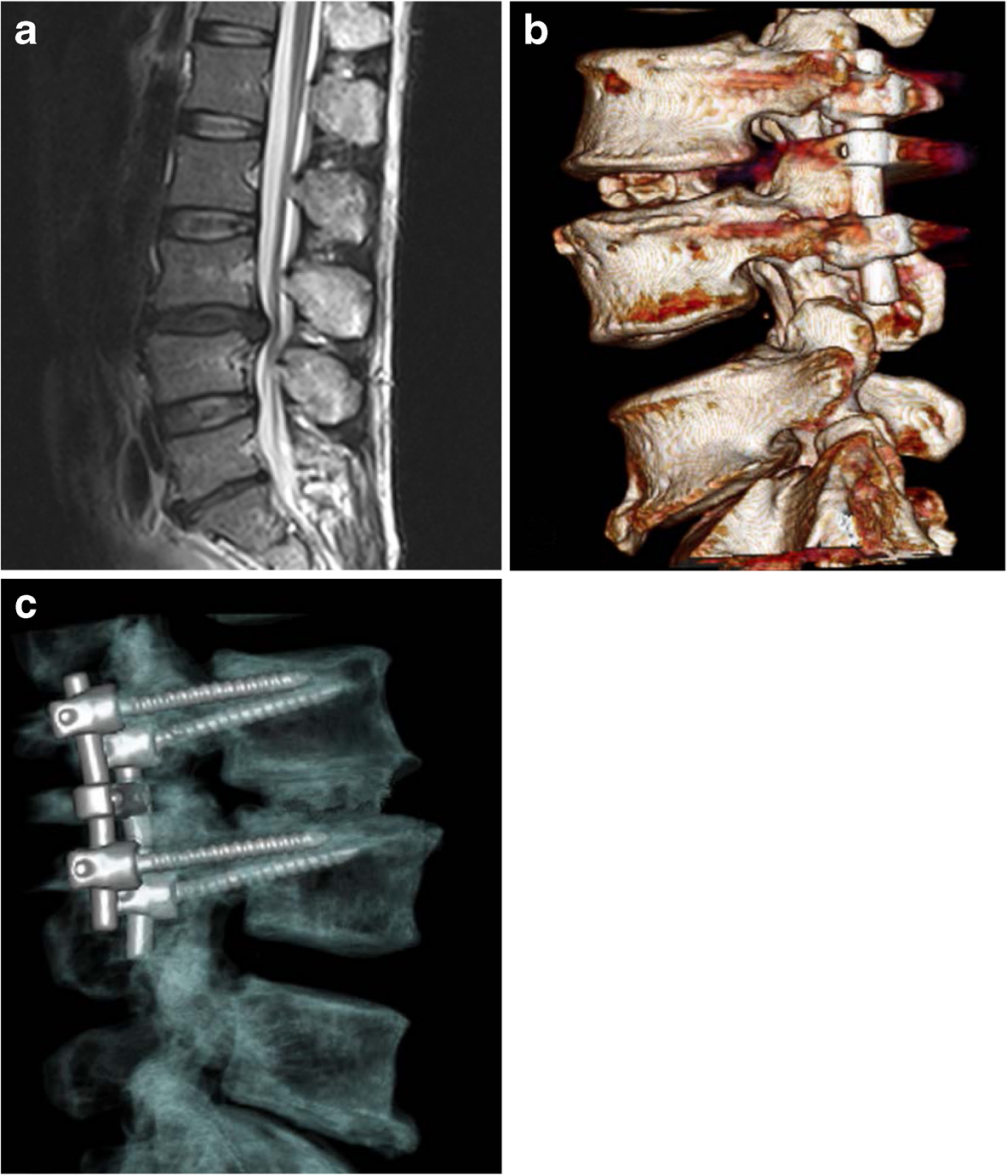
illustrates a patient who underwent an L3/L4 PLIF with the natural cage: a preoperative MRI (**a**); CT scan show the natural cage at 1 week postoperatively (**b**); CT scan at 1.5 years after PLIF (**c**), showing stable bony fusion

**Fig. 4:**
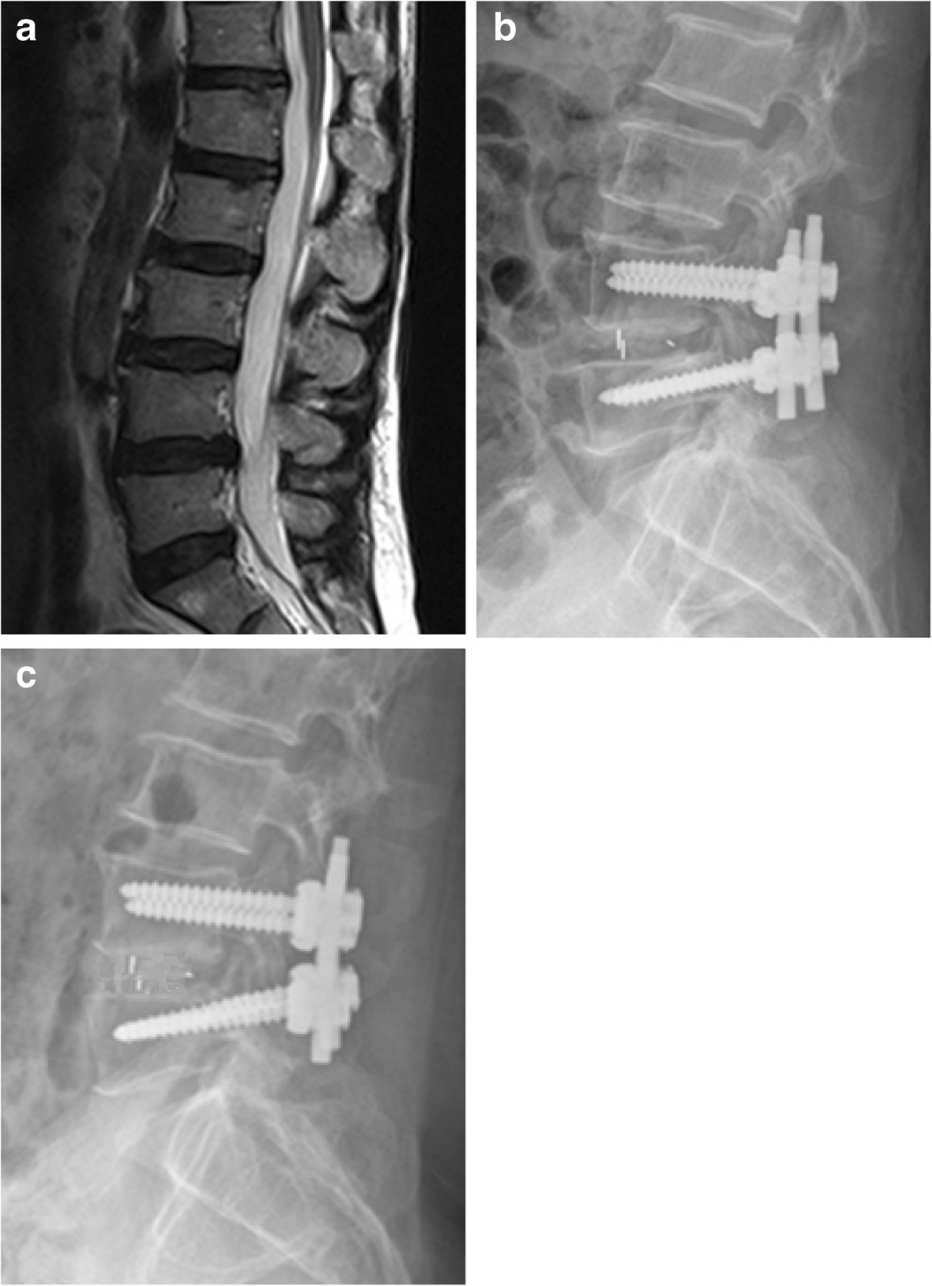
illustrates examples of a patient who underwent an L4/L5 PLIF with the PEEK cage: a preoperative MRI (**a**); lateral x-rays postoperative (**b**); and lateral radiographs at 12 months post-operatively, showing fusion at L4/L5

Overall, outcomes in both groups were satisfactory. There were no instances in either group of surgery-related neurological deficit, wound breakdown, hardware loosening or breaking, or neurological injury due to violation of the pedicle cortex by the screws. Figure 3 illustrates examples of a patient who underwent an L3/L4 PLIF with a PEEK cage. Figure 4 illustrates a patient who underwent an L3/L4 PLIF with an ACSP.

## Discussion

In the 1950s, Ralph B. Cloward, MD, developed the posterior lumbar interbody fusion using impacted blocks of bone taken from the iliac crest [6]. Since then, PLIF has been widely used for treatment of degenerative disc disease (DDD) following failure of conservative treatment [17]. Over the past several decades, the continuous modification and refinement of surgical techniques, such as minimization of the level of neural retraction required and avoidance of broad dissection of the paraspinal musculature, have contributed to a reduction in the operative risks, operating time, and blood loss during PLIF [18].

Normally, PLIF is performed using synthetic intervertebral cages with autogenous bone or another allograft implanted in the intervertebral space [7]. The surgical goals of PLIF with a cage are to provide an adequate fusion environment, thereby hastening postoperative rehabilitation and fusion [7]. Many studies have postulated that successful fusion results in better functional outcome and better overall satisfaction [19, 20]. This suggests that stabilizing the collapsing spine with successful fusion may play a significant role in the patient’s clinical improvement, even if the primary motivation for surgical intervention is adequate nerve decompression for the resolution of radicular symptoms and/or neurogenic claudication [21, 22]. Some researchers believe that once the unstable segment is successfully fused, mechanical back pain from a pars defect or facet arthropathy can be reduced, which may contribute to good functional outcomes [20, 23]. Therefore, successful lumbar fusion will most likely predict a satisfactory clinical outcome.

To achieve a solid arthrodesis in spinal fusion, a suitable graft material is needed to induce the formation of new bone at the surgical site [24]. The ideal graft for PLIF is one that will cause the least donor-site morbidity, and provide maximum efficacy of bone growth by combining osteoinduction, osteoconduction, and osteoblastic properties [25]. Autologous iliac crest bone graft (ICBG) has historically been the gold standard material for spinal fusion due to its osteogenic, osteoinductive and osteoconductive characteristics. Unfortunately, it comes with significant graft-site morbidity, with up to 30 % of patients experiencing persistent donor site-associated pain [26]. To eliminate the side effects of ICBG, many alternative materials have been explored for use as a grafting material, such as titanium cylinders, carbon fiber cages, tantalum blocks, and polyetheretherketone (PEEK). PEEK cages are currently the most often available and most widely used [25, 27, 28]. The PEEK cage can be easily sterilized and stored, significantly reducing the risk of disease transmission by allograft bone. Furthermore, the use of a PEEK cage for interbody fusion is conducive to afford immediate anterior load sharing and for restoration of disc height in situations where degeneration of the disc has caused collapse of the vertebral body [29]. Previous studies have demonstrated fusion rates comparable to historical data using existing nonresorbable implants. More importantly, there appears to be minimal risk of direct or indirect implant related adverse effects [30, 31]. Several authors have reported successful fusion in up to 90–95 % of patients undergoing PLIF with a PEEK cage [32].

Though the current analysis confirmed the high likelihood of good clinical outcomes using cages, cages still have many intrinsic disadvantages. The direct insertion of a synthetic implant reduces the available contact area for bony fusion in the fusion area. Studies have shown that greater than 30 % of the surface area of the end plate should be in direct contact with the local bone [33]. Moreover, the cage is a foreign body and may increase the patient’s risk of developing an infection or immunological problem [34]. Previous studies have reported several complications of synthetic cages, including risk for subsidence and corrosion [35]. In addition, the high cost of cages remains an obstacle, especially in developing countries.

One possible resolution to the pitfalls of nonresorbable cages and the morbidity associated with ICBG was proposed by Simmons [36] through the use of harvested corticocancellous autologous bone from the posterior elements of the vertebra being treated without a cage scaffold. However, complications such as collapse and pseudoarthrosis have been reported with these grafts [37]. To reduce the risk of these complications and still obtain good contact between the intervertebral spacers, we developed an alternative graft to the ACSP to better achieve fusion in the current study.

Fusion occurred in 32 patients (94.1 %) who received a PEEK cage and in 34 patients (97.1 %) who received an ACSP by 8–12 months post-operatively; these results are consistent with reports in the literature [38]. The mean lumbar lordosis and mean disc height to vertebral body ratio were restored and preserved postoperatively. Nevertheless, no significant differences existed between the two groups. Importantly, functional outcomes in both groups improved considerably, and there was a significant decrease in back pain comparing pre- and postoperative VAS scores in both groups. These findings indicate that both groups had similar nerve compression relief and satisfactory clinical outcomes.

Though we obtained similar clinical outcomes from both groups for single level PLIF in the present study, we believe that the ACSP has more potential advantages than the PEEK cage. The ACSP corrected lumbar lordosis and intervertebral space height to a similar extent compared with PEEK cage without the need for a bone graft from the iliac crest. Additionally, bony fusion of the vertebral body between the bone graft and the endplate was easily confirmed by CT. The ACSP is an autograft and has virtually no potential risk of foreign body reaction. Finally, the use of ASCP could reduce the cost of PLIF compared to using a PEEK cage, which could be especially appealing for use in developing countries with fewer resources.

Several points should be kept in mind in order to achieve satisfactory outcomes for PLIF using an ACSP. All soft tissue on the bone grafts should be completely removed prior to implantation, and the cage should be appropriately positioned. When inserting the remaining bone chips, the smaller portions should be introduced into the front of the intervertebral space, and the larger grafts that consist of cortical bone should be positioned in the back to restore disc height.

One limitation of this study is that the follow-up time did not extend long enough to study the long-term effects of these two different methods. Longer follow-up is needed. Our sample size was relatively small, although it exceeded the size for detecting a statistical difference in clinical outcomes. With further development of this technique, it is expected that the clinical results will become more reliable.

## Conclusion

In conclusion, satisfactory fusion rates and restoration of intervertebral height can be achieved and maintained in PLIF using either an ACSP made of bone graft taken from the patient’s spinous processes and laminae or a PEEK cage with an iliac bone graft. Our results suggest that this ACSP is as safe and effective as the PEEK cage, and can be used as an alternative means for spinal fusion in PLIF for lumbar degenerative diseases without major instability.

## Abbreviations

PLIF: Posterior lumbar interbody fusion
PEEK: Polyetheretherketone
ACSP: Autologous cage made from the lumbar spinous process and laminae
VAS: Visual Analogue Score
DH: Disc height
RL: Regional lordosis
DDD: Degenerative disc disease
